# Evaluating a Semiautonomous Brain-Computer Interface Based on Conformal Geometric Algebra and Artificial Vision

**DOI:** 10.1155/2019/9374802

**Published:** 2019-11-27

**Authors:** Mauricio Adolfo Ramírez-Moreno, David Gutiérrez

**Affiliations:** Centro de Investigación y de Estudios Avanzados (Cinvestav), Unidad Monterrey, Apodaca, Nuevo Leon 66600, Mexico

## Abstract

In this paper, we evaluate a semiautonomous brain-computer interface (BCI) for manipulation tasks. In such a system, the user controls a robotic arm through motor imagery commands. In traditional process-control BCI systems, the user has to provide those commands continuously in order to manipulate the effector of the robot step-by-step, which results in a tiresome process for simple tasks such as pick and replace an item from a surface. Here, we take a semiautonomous approach based on a conformal geometric algebra model that solves the inverse kinematics of the robot on the fly, and then the user only has to decide on the start of the movement and the final position of the effector (goal-selection approach). Under these conditions, we implemented pick-and-place tasks with a disk as an item and two target areas placed on the table at arbitrary positions. An artificial vision (AV) algorithm was used to obtain the positions of the items expressed in the robot frame through images captured with a webcam. Then, the AV algorithm is integrated into the inverse kinematics model to perform the manipulation tasks. As proof-of-concept, different users were trained to control the pick-and-place tasks through the process-control and semiautonomous goal-selection approaches so that the performance of both schemes could be compared. Our results show the superiority in performance of the semiautonomous approach as well as evidence of less mental fatigue with it.

## 1. Introduction

A brain-computer interface (BCI) is a system that enables a real-time user-device communication pathway through brain activity. Through the years, research and development on BCI has mainly been oriented to the creation of rehabilitation systems as well as systems that help disabled patients regain to some extent their lost or diminished capabilities [1]. Some reported devices that have been successfully controlled using BCIs are spellers, electric wheelchairs, robotic arms, electric prostheses, and humanoid robots [2–5]. In BCI studies, the most common technique used to acquire brain noninvasively is electroencephalography (EEG).

In order to manipulate the device through brain activity, the design of the BCI must include the following stages: signal acquisition, filtering, feature extraction, classification, device modeling, and control [6]. During the filtering stage, unwanted noise and artifacts are removed from the signals using temporal and spatial filters. Then, temporal or spatial features of interest are extracted from the signals to build feature vectors. These vectors are formed by characteristic components of the signals, which are then used in the classification stage to decipher user intention. Lastly, the device is manipulated based on the result of the classification algorithm. Depending on the device and the complexity of the system, a model of the system is needed to perform with precision the desired tasks. BCIs can be divided into two groups based on their control strategy: *process control* and *goal selection*. In the process-control strategy, users are continuously controlling each part of the process. This is done by performing low-level commands continuously through the BCI, with no additional assistance. On the other hand, in the goal-selection strategy, users are responsible for selecting their desired goal and the system provides assistance to successfully perform the tasks with minimum effort [7]. In this case, the user performs high-level tasks by sending simple commands through the BCI.

Common paradigms used as control commands in BCI include steady-state visual evoked potentials (SSVEPs), P300 waveform, and motor imagery (MI). SSVEP is a resonance phenomenon occurring at occipital and parietal lobes as a result of oscillatory visual stimulus presented to a user at a constant frequency [8]. The P300 is an EEG signal component that appears 300 ms after an event of voluntary attention, and it is usually observed during visual or auditory stimulus presentation [9]. MI presents as an event-related desynchronization (ERD) found at the sensorimotor areas, which generates a contralateral power decrease in a frequency range from 8–13 Hz (also known as the *μ* band) [10]. Controlling a BCI with SSVEP and P300 requires less training in comparison to MI, as the first represents an involuntary response to a stimulus. However, its use in BCI is limited due to its requirement of a stimulus presentation device. The training process to control MI-based BCIs (MI-BCI) might involve stimulus presentation as well. However, it can be excluded for its final application on the BCI. Even though MI-BCIs require longer training periods, they are better suited for close-to-real-life environments and self-paced BCIs [11].

Several studies present successfully implemented ERD-based BCIs, most of them using a process-control strategy [12–14]. Some goal-selection BCIs have been reported as well [15, 16]. In [7], users were trained on process-control and goal-selection MI-BCIs to perform one-dimensional cursor movements on a screen. The results suggest that users performing on goal-selection strategy showed higher accuracy and faster learning in comparison to the process-control approach. However, the authors state that a direct comparison of goal selection and process control in a more complicated (real-world) scenario has not yet been presented. In the proposed study, three-dimensional object manipulation tasks through a robotic arm are implemented in a MI-BCI. The complexity of the three-dimensional movements on real objects is higher than the one-dimensional movements on virtual objects presented in [7]. In [17], a semiautonomous BCI is implemented to manipulate a robotic arm to perform tasks such as pouring a beverage inside a glass in a tray, through SSVEP. In future research, similar tasks as in [17] could be implemented in our BCI using MI instead, allowing a more natural execution of daily-life context tasks without the need of a stimulus presentation screen.

In a typical process-control MI-BCI, the user controls the direction of the final effector of a robotic arm through low-level commands, which means that the user has to maneuver the robot in a three-dimensional space to reach for a desired target. Clearly, the user remains in a high attention state during the maneuvers, as he/she is continuously aware of the final effector position during the whole task. This continuous awareness might lead to generation of mental fatigue or frustration, which is undesirable as it can directly affect user performance and learning [18]. The analysis of P300 features, such as amplitude and latency, has been shown to be useful in identifying the depth of cognitive information processing [19]. The amplitude of P300 waveform tends to decrease when users encounter cognitive tasks with high difficulty [20]. On the other hand, P300 latency has shown to increase when the stimulus is cognitively difficult to process [21]. Another study has reported correlation between changes in the P300 component and BCI performance [22]. The evidence provided by these studies might suggest that the analysis of P300 could be implemented as a mental fatigue indicator during BCI training and control.

In order to diminish mental fatiguing in BCI systems, a semiautonomous BCI using a goal-selection strategy is here proposed. This system assists the user to perform a specific task by calculating all the variables needed to successfully execute it. Some studies have previously presented BCI designs focusing on this semiautonomous approach with successful results on performance, accuracy, and comfort for the user [17, 23, 24]. Therefore, this paper presents the implementation of a traditional low-level MI-BCI and a semiautonomous MI-BCI designed to perform object manipulation tasks with a robotic arm. In the process-control strategy MI-BCI, the user commands the final effector of the robot to move in a three-dimensional space to reach for a target placed on a table. In the semiautonomous MI-BCI, one small disk and two target areas are placed on a table. Here, the robot reaches for the disk and places it on a specific target, which is selected by the user. As proof-of-concept, two volunteers were trained on each BCI system, and their performance was evaluated and compared. A statistical P300 analysis was performed on all users in order to observe mental fatigue differences induced by the operation of low-level and semiautonomous BCIs.

In order to model the robot used in this experiment, a conformal geometric algebra (CGA) model was implemented in both the traditional and semiautonomous BCIs to solve the inverse kinematics of the robotic arm, i.e., obtaining the joint angles needed for a specific position of the final effector. Additionally, an artificial vision (AV) algorithm was integrated into the semiautonomous BCI in order to provide information about the positions of the items on the table referenced to robot frame. As the implementation of the semiautonomous BCI implies a higher computational load, the CGA model was chosen for the solution of the inverse kinematics. CGA has shown to represent an operation reduction and in some cases, a decrease in computational load when compared to traditional inverse kinematics solution [25].

This paper is organized as follows. The CGA model and AV algorithm are described in Section 2, and the design of both BCIs is explained in Section 3. Evaluations on both algorithms and performance results of users controlling both BCIs are presented in Section 4. Preliminary short reports of the system's implementation (but not its evaluation) have been presented in [26] and [27].

## 2. Robot Modeling and Artificial Vision

In this section, we describe each of the components required to compute the inverse kinematics of a robotic arm by using CGA. Furthermore, here we explain in detail the AV algorithm used to obtain the positions of the objects to be manipulated by the robot.

### 2.1. Conformal Geometric Algebra

Traditional methods to solve the inverse kinematics of robots include several matrix operations as well as many trigonometric expressions. All this can result in a quite complex solution depending on the modeled robot [28]. In this study, a conformal geometric algebra (CGA) model is proposed instead, as it is considered to be computationally lighter, easier to implement, and highly intuitive. CGA has proved to be a powerful tool when solving the inverse kinematics of robotic arms [29, 30]. It also offers an operation reduction when compared to traditional methods and provides efficient runtime solutions. More information on computational efficiency characteristics can be found in [31].

With this model, the joint angles of the robot are obtained for a specific position of the final effector. In CGA, two new dimensions (*e*_0_, *e*_*∞*_) are defined, representing a point in the origin and a point in the infinity, respectively, in addition to the three-dimensional Euclidean space (*e*_1_, *e*_2_, *e*_3_) [29]. In this space, geometric entities (points, lines, circles, planes, and spheres) and calculations involving them (distances and intersections) can be represented with simple algebraic equations.

Also, the geometric product between two vectors *a* and *b* is defined as a combination of the inner product and the outer product:

(1) $$\begin{array}{c} ab=a\cdot b+a\wedge b. \end{array}$$

The inner product is used to calculate distances between elements, and the outer product generates a bivector, which is an element occupying the space spanned by both vectors. It is also used to find the intersection between two elements. The intersection *M* of two geometric objects *A* and *B* represented in CGA is given by *M* ^*∗*^=*A* ^*∗*^∧*B* ^*∗*^ or *M* ^*∗*^=*A* ^*∗*^ · *B*. The element *A* ^*∗*^ is the duality of *A* and is expressed as

(2) $$\begin{array}{c} A^{*}=AI_{c}^{-1}, \end{array}$$

where *I*_*c*_^−1^=*e*_0_ *e*_3_ *e*_2_ *e*_1_ *e*_*∞*_, which allows for a change in representation of the same element. Standard and dual representations of commonly used geometrical objects in CGA are shown in Table 1. There, *x* and *n* are points represented as a linear combination of the 3D base vectors:

(3) $$\begin{array}{c} x=x_{1}e_{1}+x_{2}e_{2}+x_{3}e_{3}. \end{array}$$

There are two possible representations of the same element, as shown in Table 1. A circle can be represented as the space spanned by three points in space as well as the intersection of two spheres. Also, a line can be expressed as the intersection of two planes as well as the space spanned by two points expanded to the infinity.

**Figure 1:**
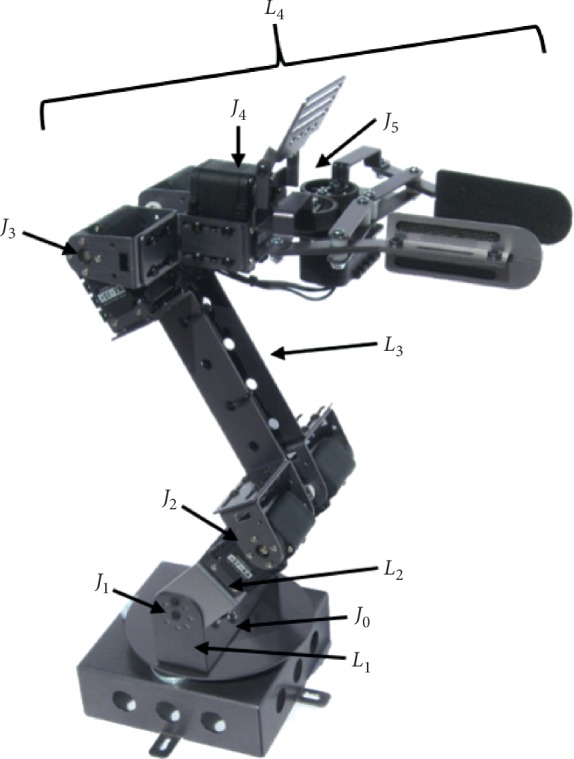
Joints and links of our 5-DOF Dynamixel AX-18A Smart robotic arm.

Making use of the previous equations and relationships, a CGA model to solve the inverse kinematics of a manipulator robot was obtained following the proposed method in [32]. The modeled robot was the Dynamixel AX-18A Smart robotic arm, which is a five-degree-of-freedom (5-DOF) manipulator robot.  Figure 1 shows the modeled robot as well as its joints and links. The DOF of this robot corresponds to its shoulder rotation, elbow flexion-extension, wrist flexion-extension, wrist rotation, and hand open-close function [33]. The inverse kinematics solution was obtained for joints *J*_0_, *J*_2_, and *J*_3_. For the particularities of the manipulation tasks, joints *J*_4_ and *J*_5_ were not considered for simplicity.

### 2.2. Our CGA Model

Next, we describe the required CGA model that we implemented specifically for our system.

#### 2.2.1. Fixed Joints and Planes

**Figure 2:**
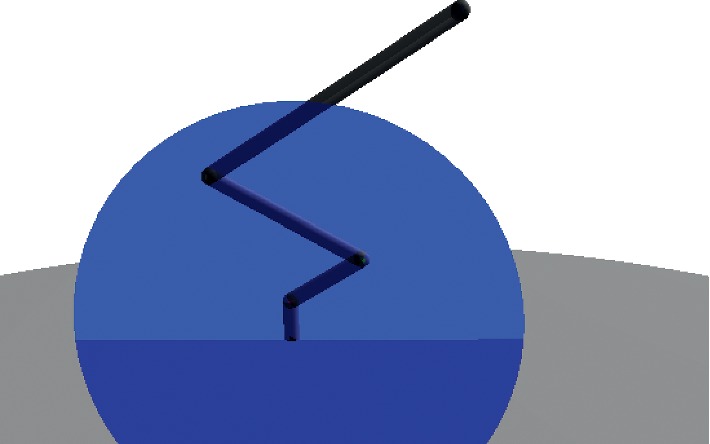
Planes *π*_*e*_ and *π*_*b*_ representing the orientation of the final effector and the robot base, respectively.

The origin of the CGA model was located at joint *J*_0_, located at the center of the rotational base of the robot; therefore, *J*_0_=*e*_0_. Joint *J*_1_ is also a fixed joint with constant position, found directly above joint *J*_0_. The position for joint *J*_1_ was defined as *x*_1_=[0,0,0.036]. Now, let us consider the desired final effector position as a point in space *x*_*e*_. Then, a vertical plane *π*_*e*_ representing the direction of the final effector is described as

(4) $$\begin{array}{c} \pi_{e}=e_{0}\wedge e_{3}\wedge x_{e}\wedge e_{\infty }, \end{array}$$

where *e*_0_ represents the origin in robot frame and *e*_3_ the Euclidian *z* axis. As the position of the final effector is used to define *π*_*e*_, the direction of plane changes consistently with *x*_*e*_. A plane *π*_*b*_, representing the rotational base of the robot, is defined as

(5) $$\begin{array}{c} \pi_{b}=e_{0}\wedge e_{1}\wedge e_{2}\wedge e_{\infty }, \end{array}$$

where *e*_1_ and *e*_2_ represent the Euclidean *x* and *y* axes. Planes *π*_*e*_ and *π*_*b*_ are shown in Figure 2.

#### 2.2.2. Calculation of Joint's Position

In a kinematic chain model of a robotic arm using CGA, the implemented method to find joint *J*_*n*_ is based on the intersection of two spheres centered at joints *J*_*n*−1_ and *J*_*n*+1_ with radii equal to the lengths of the links connecting *J*_*n*−1_ with *J*_*n*_ and *J*_*n*_ with *J*_*n*+1_, respectively. The intersection of both spheres results in a circle, which is then intersected to the plane of the final effector to obtain a point pair representing two possible configurations for joint *J*_*n*_. One point is then selected as *J*_*n*_, depending on the desired configuration. The process requires the following:

(i) Spheres centered at point *P* with radius *r* are given by
  (6) $$\begin{array}{c} s=P-\frac{1}{2}r^{2}e_{\infty }. \end{array}$$

(ii) There are two methods for creating a circle. We can either intersect two spheres *s*_*j*_ and *s*_*k*_ by
  (7) $$\begin{array}{c} c={\left(s_{j}^{*}\wedge s_{k}^{*}\right)}^{*}, \end{array}$$

or we can intersect a plane *π* and a sphere *s* by
  (8) $$\begin{array}{c} c={\left(\pi^{*}\wedge s^{*}\right)}^{*}. \end{array}$$

(iii) The intersection of a circle *c* and a plane *π* to create a point pair *Pp* is given by
  (9) $$\begin{array}{c} Pp={\left(c^{*}\wedge \pi^{*}\right)}^{*}. \end{array}$$

(iv) Finally, to obtain a point *P* from *Pp*, we have
  (10) $$\begin{array}{c} P=\frac{Pp\pm \sqrt{Pp^{2}}}{-e_{\infty }\cdot Pp}. \end{array}$$

Based on the previous expressions, and in order to find the position of joint *J*_2_ in our modeled robot, two spheres must be constructed, and they have to be centered at *J*_1_ and *J*_3_. However, the position of joint *J*_3_ is yet unknown in our model. A similar situation occurs if the desired position is instead joint *J*_3_. In this particular case, *x*_*e*_ is known but not *J*_2_. Given this situation, another approach was implemented in order to find joint *J*_2_.

#### 2.2.3. Position of Joint *J*_2_

Using (6), sphere *s*_1_ was centered at *x*_1_ with radius equal to the length of link *L*_2_. Hence, in order to find joint *J*_2_, another sphere *s*_*h*_ must be intersected to *s*_1_. In order to construct *s*_*h*_, its center must be defined. This is achieved by first creating an auxiliary sphere *s*_0_, centered at the origin with radius *L*_*a*_ equal to the horizontal component of the distance from *J*_0_ to *J*_2_. This is valid as the distance from *J*_0_ to *J*_2_ is constant for any position of the final effector *x*_*e*_.

**Figure 3:**
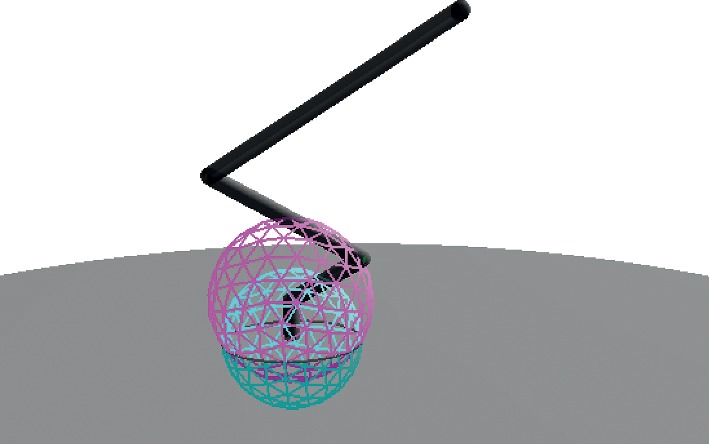
The intersection of spheres *s*_0_ (bottom) and *s*_1_ (top) define the circle *c*_0_, from which we find the position of point *x*_*h*_.

Then, using (8), *s*_0_ is intersected to plane *π*_*e*_ to obtain circle *c*_0_. Next, using (9), *c*_0_ is intersected to plane *π*_*b*_ to produce point pair *Pp*_0_, from which one point is selected as *x*_*h*_ using (10). The procedure to find point *x*_*h*_, which corresponds to the center of the desired sphere to be intersected with *s*_1_, is shown in Figure 3.

**Figure 4:**
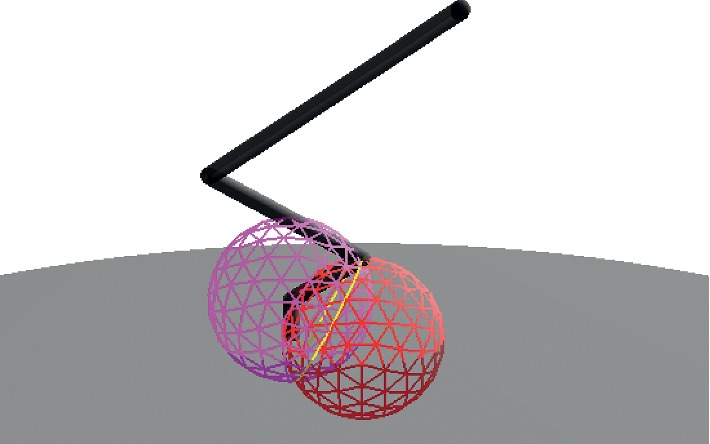
The intersection of spheres *s*_1_ (left) and *s*_*h*_ (right) define the circle *c*_2_, from which we find the position of joint *J*_2_.

Using (6), sphere *s*_*h*_ is centered at *x*_*h*_ with radius *L*_*b*_ equal to the vertical component of the distance from *J*_0_ to *J*_2_. Then, the intersection of spheres *s*_1_ and *s*_*h*_ is given by (7), which results in circle *c*_2_. Using (9), the intersection of *c*_2_ with plane *π*_*e*_ renders point pair *Pp*_2_. Finally, the position of *J*_2_ is obtained from *Pp*_2_ given by (10). The whole procedure previously detailed to obtain the position of joint *J*_2_ is represented in Figure 4.

#### 2.2.4. Position of Joint *J*_3_

**Figure 5:**
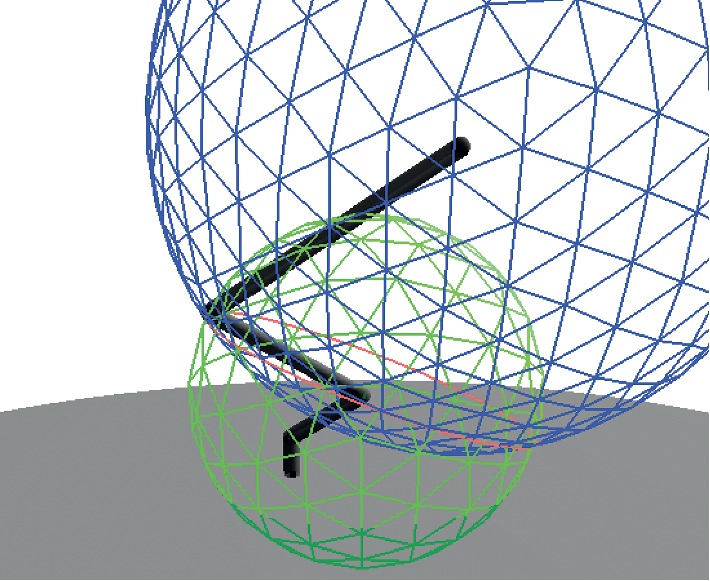
The intersection of spheres *s*_2_ (bottom) and *s*_*e*_ (top) define the circle *c*_3_, from which it is possible to find the position of joint *J*_3_.

The procedure to find the position of joint *J*_3_ is straight forward once the position of joint *J*_2_ is calculated. For that, two spheres *s*_2_ and *s*_*e*_ are defined using (6), centered at *x*_2_ and *x*_*e*_, with radii equal to the length of links *L*_3_ and *L*_4_, respectively. Both spheres are intersected to obtain circle *c*_3_ using (7). With (9), *c*_3_ is then intersected to plane *π*_*e*_ to obtain point pair *Pp*_3_. From *Pp*_3_, *J*_3_ is easily obtained using (10). A representation of the procedure to find joint *J*_3_ is shown in Figure 5.

#### 2.2.5. Angle Calculation

In order to calculate the angles formed by two vectors *α* and *β*, their corresponding unit vectors are defined as $\hat{\alpha }=\alpha /\left\|\alpha \right\|$ and $\hat{\beta }=\beta /\left\|\beta \right\|$. The normalized bivector spanning the space formed by those vectors is expressed as

(11) $$\begin{array}{c} \hat{N}=\pm \frac{\hat{\alpha }\wedge \hat{\beta }}{\left\|\hat{\alpha }\wedge \hat{\beta }\right\|}. \end{array}$$

As explained in [32], the angle *θ* between *α* and *β* is given by

(12) $$\begin{array}{c} \theta =A\;\tan\;2\left[\frac{\left(\alpha \wedge \beta \right)}{\hat{N}},\alpha \cdot \beta \right], \end{array}$$

where *A* tan 2 corresponds to the *four-quadrant* inverse tangent. This operator gathers information on the signs of its two arguments in order to return the appropriate quadrant of the computed angle [34]. Such result is not possible to be obtained from the conventional single-argument arctan function. Also, note that the plus sign in (11) applies if the rotation from *α* to *β* is counter-clockwise, while the minus sign applies in the opposite rotation.

In order to find the joint angles using (12), vectors formed by the links of the robot need to be calculated. First, lines representing each link are defined:

(13) $$\begin{array}{c} l_{01}=e_{0}\wedge J_{1}\wedge e_{\infty },\\ l_{12}=J_{1}\wedge J_{2}\wedge e_{\infty },\\ l_{23}=J_{2}\wedge J_{3}\wedge e_{\infty },\\ l_{3e}=J_{3}\wedge J_{e}\wedge e_{\infty }. \end{array}$$

The previous expressions define lines passing through links *L*_1_, *L*_2_, *L*_3_, and *L*_4_, respectively (see Figure 1). *L*_4_ was considered a straight line from joint *J*_3_ to the final effector *x*_*e*_, i.e., we ignored wrist rotation and hand open-close joints.

In (12), the parameters *α* and *β* need to be *directional* vectors for the purpose of computing our joint angles. Therefore, the directional vectors of plane *π*_*e*_, as well as lines *l*_23_ and *l*_3*e*_, were calculated, which represent the base and links of the robot, respectively. From a given line *l*, its directional vector can be obtained as

(14) $$\begin{array}{c} \left(l\cdot e_{0}\right)\cdot e_{\infty }, \end{array}$$

and the directional vector normal to a plane *π* is given by

(15) $$\begin{array}{c} \left(\pi^{*}\wedge e_{\infty }\right)\cdot e_{0}. \end{array}$$

Based on all the previously defined elements, the vectors involved in the calculation of joint angles *θ*_*k*_, for *k*=0,2,3, are summarized in Table 2. Then, *α* and *β* in (11) and (12) are replaced by *α*_*k*_ and *β*_*k*_, respectively, to calculate *θ*_*k*_. Note that, as joint *J*_1_ is fixed, *θ*_1_ does not need to be calculated.

### 2.3. Artificial Vision Algorithm

An AV algorithm was implemented to calculate the positions of items on a table, so the robotic arm could perform the desired manipulation tasks. An ATW-1200 Acteck web camera was used to record images at 30 fps with a resolution of 640 × 480 pixels. The acquired images were processed and analyzed in real time using the OpenCV library (https://www.opencv.org) from Python.

**Figure 6:**
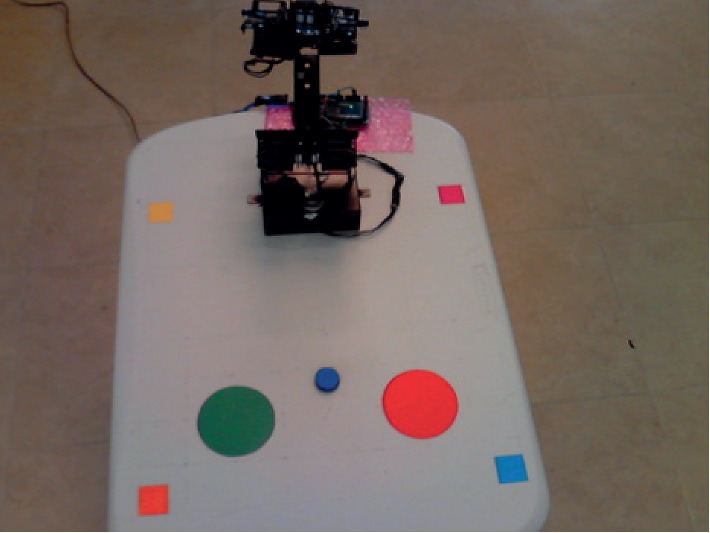
Robot and items on the table as seen by the camera during semiautonomous BCI trials.

The robotic arm was fixed on a white table, centered at one end of it. A plane was delimited on the table, defined as 400 × 400 mm^2^. Four 30 × 30 mm^2^ markers of different colors (cyan, orange, magenta, and yellow) were placed inside the delimited square, one at each corner. A blue disk with height of 6 mm and radius of 13 mm was used as the item to be picked, while two stickers with radius of 42 mm (green and red) were used to indicate target areas. The camera was fixed in a high angle so that all markers and items were inside its field of view. The setup of the robotic arm and items in the table are shown in Figure 6.

In order to perform object manipulation tasks, the *real-world* coordinates of the plane (in reference to robot frame) had to be obtained from the *image* coordinates obtained by the camera. To achieve this, a homography transformation was performed on the acquired images. In general, a two-dimensional point (*u*, *v*) in an image can be represented as a three-dimensional vector (*x*, *y*, *z*) by letting *u*=*x*/*z* and *v*=*y*/*z*. This is called the homogeneous representation of a point, and it lies on the projective plane *P* ^2^ [35]. Homography is invertible mapping of points and lines on the projective plane *P* ^2^, thus allowing to obtain the real-world coordinates of features in an image from its image coordinates.

In our case, the desired transformation is such that the image obtained from the camera is turned into a two-dimensional view of the same setup. In this transformation, the image shows a planar representation of the original view, as if the camera was placed directly above the delimited square. In order to obtain this representation, the following homography transformation was applied [35]:

(16) $$\begin{array}{c} \left[\begin{array}{c} u\\ v \end{array}\right]=H\left[\begin{array}{c} x\\ y \end{array}\right], \end{array}$$

where vectors [*uv*]^*T*^ and [*xy*]^*T*^ represent the positions of selected points in the image and their corresponding positions in real-world coordinates, respectively, *H*=*K*[*R*|*t*] is the homography matrix that defines the desired perspective change to be performed on the image, and *K* is the calibration matrix which contains the intrinsic parameters of the camera, while *R* and *t* are, respectively, the rotation matrix and translation vector applied on the camera in order to perform this transformation view. In (16), *z* is ignored as all items are considered to be at *z*=0.

**Figure 7:**
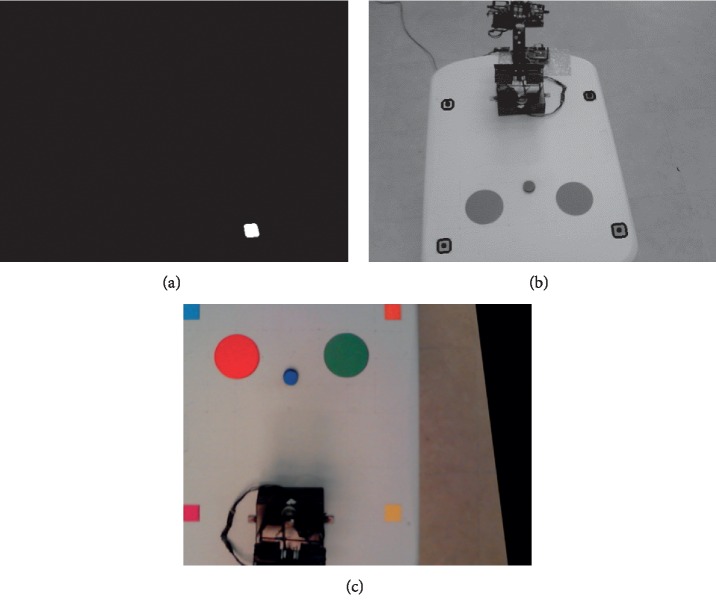
Required steps of the AV algorithm. (a) Segmentation and binarization. (b) Centroid calculation. (c) Homography transformation.

In order to compute matrix *H*, both real-world and image coordinates of the centroids of the square markers were obtained. First, markers were detected through color segmentation and binarization, as shown in Figure 7(a). This process was performed separately on each marker, and their contours were detected. After that, the centroids of the markers in the image were calculated. The contours and centroids of each marker are shown in Figure 7(b).

Since the markers have known dimensions (30 × 30 mm), the positions of their centroids in real-world coordinates relative to the plane are known as well. These positions were defined as cyan at [15,15] mm, orange at [385,15] mm, magenta at [15,385] mm, and yellow at [385,385] mm, all inside the available 400 × 400 mm area of the table. Then, both sets of coordinates are used to obtain *H* with OpenCV's command *findHomography*, and the resulting matrix is applied to transform the image, as shown in Figure 7(c).

Then, using the same procedure as with the markers, the centroids of the disk and targets in the new image were calculated. However, the reference frame from the image is different from the reference frame from the robot. Therefore, the first was transformed by applying the following rotation matrix:

(17) $$\begin{array}{c} R=\left[\begin{array}{cc} \cos\;\pi & -\sin\;\pi \\ \sin\;\pi & \cos\;\pi \end{array}\right]. \end{array}$$

**Figure 8:**
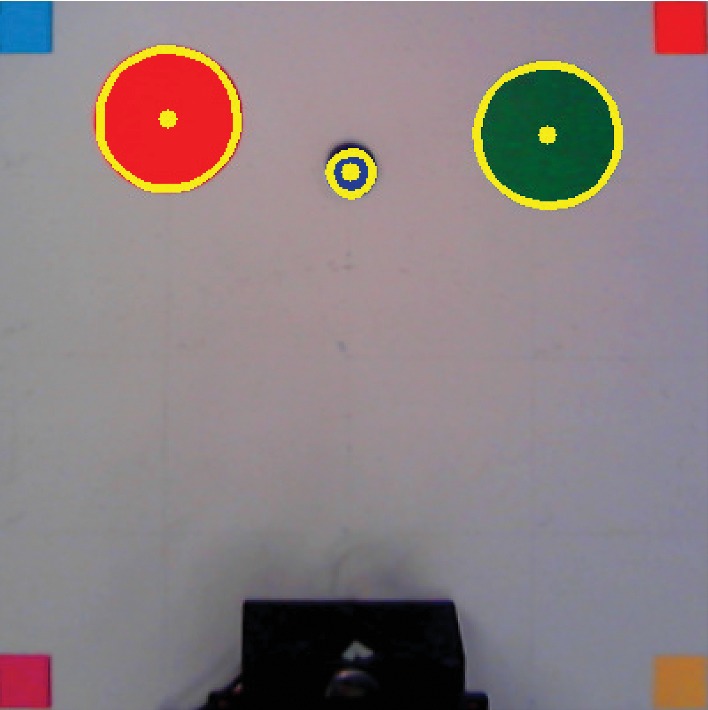
Visual representation of contours and centroids of the items in the table calculated by the AV algorithm in order to obtain their real-world coordinates.

Furthermore, a translation vector [−200 − 400]^*T*^ was applied as well as a sign switching of the *x* axis to obtain the desired positions. In robot frame, the *x* axis of the delimited square goes from −20 to 20, while the *y* axis goes from 0 to 40, and the robot is located at the origin. After applying all those transformations, the centroids of all items are finally expressed in robot frame, and they can be detected by the AV system together with the contours of all items. This is shown in Figure 8.

## 3. Implementation of BCI Systems

As proof-of-concept, four participants volunteered in this study (two females and two males, with average age of 22.25 years, SD=±0.95). The experimental protocol was divided into three stages for both the process-control and goal-selection BCIs: (i) training, (ii) cued manipulation, and (iii) uncued manipulation. Both BCIs were MI-based; therefore, users were trained to control the corresponding *μ* band desynchronization at will. In all trials, volunteers sat in front of a computer screen first showing a black screen (baseline) in which the user was meant to be in a resting state. Then, different types of stimulus were presented to the user, representing each a different command. The duration for the baseline (15 seconds) and stimulus presentation (4 seconds) was the same for all trials and stages. During stimulus presentation, users were expected to react accordingly, either by imagining the movement of either left or right hand, or by remaining in a resting state. In training trials, EEG signals were acquired and analyzed offline to build and evaluate the performance of classifiers, which were then used online during the manipulation trials. In cued manipulation trials, the user was expected to manipulate the device as indicated by the stimuli. On the other hand, the user was encouraged to manipulate the device at will during uncued manipulation trials.

### 3.1. Training Trials

The training protocol was identical for both the process-control and goal-selection BCIs. Three types of stimuli were presented to the user: *right hand imaginary movement* (RHIM), *left hand imaginary movement* (LHIM), and *rest*. A total of 30 stimuli (10 for each command) were randomly presented to the user. Stimuli were represented in the computer screen with a red arrow pointing to the right (for RHIM), pointing to the left (for LHIM), and a black screen for rest. A 2 s green cross appeared before all stimuli as a prestimulus, and there was a variable interstimulus resting period of 2–4 seconds between stimuli. Users underwent three training sessions on different days, each comprising five repetitions of the mentioned experimental protocol, while EEG recordings were obtained.

### 3.2. Signal Acquisition

EEG signals were recorded with the Mobita equipment from TMSi systems, using a measuring cap of 19 channels: FP1, FP2, F3, F4, C3, C4, P3, P4, O1, O2, F7, F8, T3, T4, T5, T6, Cz, Fz, and Pz. Impedance of all electrodes was set below 5 kΩ for all experiments. Signals were acquired with a sampling frequency of 1000 Hz. Recordings were band-pass filtered with a fourth-order 1–100 Hz Butterworth filter and a 60 Hz notch filter to eliminate power line interference. The OpenViBE software was used for the BCI design and implementation. More information about this software can be found in [36].

### 3.3. Classification Algorithm

**Figure 9:**
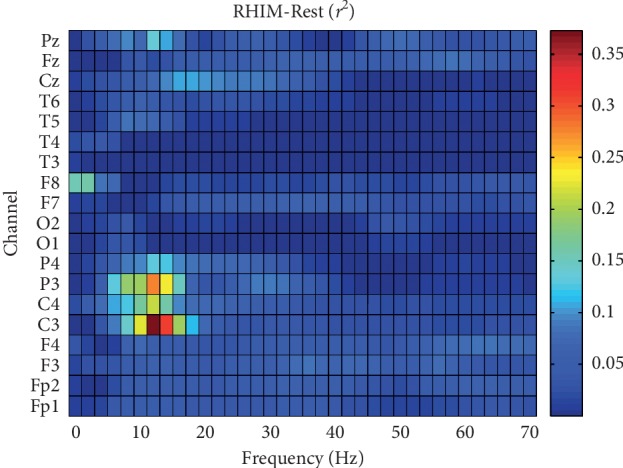
Representative *r* ^2^ map obtained during one training session. *r* ^2^ values shown here were measured under conditions RHIM-Rest for all channels and frequencies.

Feature extraction was performed using the BCI2000 offline analysis tool (https://www.bci2000.org/mediawiki/index.php/User_Reference:BCI2000_Offline_Analysis), where the *r* ^2^ value was calculated. A higher *r* ^2^ value is related to a higher discrimination of a signal under two stimulus conditions. More details about the statistic meaning of *r* ^2^ can be found at https://www.bci2000.org/mediawiki/index.php/Glossary. After each training session, signals from the five training trials were used to calculate *r* ^2^. Three *r* ^2^ maps (one per stimulus combination) were obtained per training session, showing the *r* ^2^ values in the 19 available channels and frequencies ranging from 1 to 70 Hz. Each map, as the one shown in Figure 9, represents the channels and frequencies which, for a specific combination of conditions, showed higher discrimination. Through this procedure, the selected channels and frequencies were used as features for the classification algorithm.

Signals were spatially filtered using a Laplacian filter on the selected channels as well as through a fourth-order Butterworth band-pass filter tuned to the selected frequencies. Power values were then obtained from the filtered signals to build the feature vectors, which then became the input for a linear discriminant analysis (LDA) classifier, which separates data representing different classes by finding a hyperplane which maximizes the distances between the means of the classes, while minimizing the variance within classes [37].

In our case, three pairwise classifiers per training session were obtained using this procedure: LHIM versus RHIM, LHIM versus rest, and RHIM versus rest. The three classifiers were tested online on the recorded signals to evaluate their performance as a percentage of correctly classified stimuli. The classification was performed on each four-second stimulus, divided into overlapped subepochs using a window function. Each four-second epoch was formed by 64 subepochs of two seconds, separated by 0.0625 seconds. One pairwise classifier labeled each subblock as one of the two possible classes, and the four-second epoch was classified as the mode of the classification result for all its subblocks. Then, one general classifier was built, based on the results of the three pairwise classifiers. Here, the four-second epoch of each stimuli was labeled as class *I*=1,2,  or 3 (LHIM, rest, or RHIM, respectively), if two out of the three pairwise classifiers labeled the same epoch identically. The mean performance of the general classifiers across trials is shown in Table 3 for all subjects and training sessions as well as their selected features. After training sessions, each user proceeded to perform the subsequent trials using the classifier with the highest performance obtained at the last training session.

### 3.4. Process-Control BCI

The process-control BCI was designed in such a manner that users were able to perform three-dimensional movements to complete reaching tasks. In this system, the position of the final effector as well as the desired axis in which the effector moves can be controlled through low-level MI-based commands. To achieve this, the user has two choices: moving along a selected axis (*y*-axis at the initial step) or change between axes. In the design of this BCI, the classification of a LHIM results in a −10 mm displacement, while the classification of a RHIM results in a +10 mm displacement on the selected axis. The classification of a rest event holds the position of the final effector with no displacement. The consecutive classification of two rest events in a row allowed the user for a change of axis. This change of axis takes place in the following sequence: *y*⟶*z*, *z*⟶*x*, and *x*⟶*y*.

#### 3.4.1. Cued Manipulation

**Figure 10:**
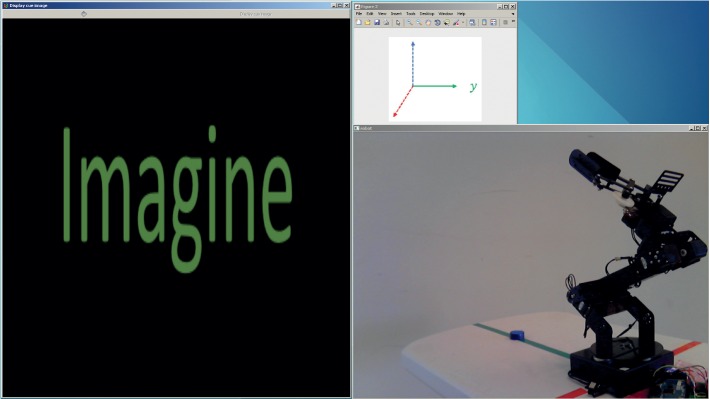
Setup of the process-control BCI. The windows shown in the screen are used for visualization of stimuli, indicating the current axis of the movement, and viewing of the robot performing the manipulation tasks.

In these trials, users sat in front of a computer showing three windows on the screen. The first window was used for stimulus presentation, the second was used to display in which axis the movement of the robot took place, and the third was used to visualize the robot and its movements. The setup for these experiments is shown in Figure 10. After the baseline period, 15 random stimuli (5 for each type) were presented to the user. Prestimulus, stimulus, and interstimulus duration was the same as in training trials (see Section 3.1). After the stimulus was presented, the user was expected to emit the instructed command through the BCI. Then, the robot performed a specific movement based on the classification result. In these trials, performance was evaluated as the percentage of correctly classified stimuli. The intention of these trials was to get the users acquainted with the BCI, and they were performed immediately before the uncued manipulation trials. Users performed three sessions on different days, each formed by three repetitions of this protocol.

#### 3.4.2. Uncued Manipulation

The same screen display was used as in cued trials, but here subjects were asked to complete reaching tasks on their own. At the start of each trial, the final effector was fixed at home position [0,155.5, 284.3] and a target was placed at [0,300, −49]. At this initial step, the distance of the final effector to the target was 360 mm. Note that the target is placed at *z*=−49, as the robot base is 49 mm above the table. A baseline period was followed by the presentation of 20 stimuli showing the word “Imagine,” in which the user was expected to emit MI commands through the BCI. The duration of prestimulus, stimulus, and interstimulus periods was the same as in training trials (see Section 3.1). The user was instructed to move the final effector as close as possible to the target within the 20 stimuli, using the protocol described in Section 3.4. Performance was evaluated as the percentage of stimuli where the user moved the final effector closer to the target and changed successfully to the *y*-axis. Users performed three sessions on different days, each formed by five repetitions of the described protocol.

### 3.5. Goal-Selection BCI

The goal-selection BCI was designed to perform in a semiautonomous way pick-and-place tasks with the disk and two possible targets. Users were able to perform these tasks for any position of the items (randomly chosen before a trial), inside of the robot workspace. The centroids *C*=[*C*_*x*_, *C*_*y*_] of the two target stickers were calculated in these trials by the AV algorithm. In this case, the classification of three types of events resulted in different manipulation tasks:

- If an event was classified as RHIM, the robot reached for the disk, placed it on the target located to the right (greater *C*_*x*_ component), and returned to home position
- If an event was classified as LHIM, the robot reached for the disk, placed it on the target located to the left (smaller *C*_*x*_ component), and returned to home position
- If an event was classified as rest, the robot remained at home position

After the robot performed a manipulation task, all the items in the table were manually changed to random positions, in preparation for the next trial.

#### 3.5.1. Cued Manipulation Trials

**Figure 11:**
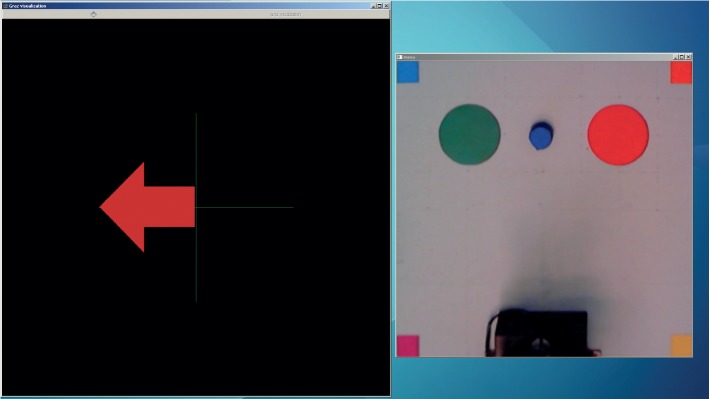
Setup of the semiautonomous goal-selection BCI, as seen by the user. The windows are used for stimulus presentation and visualization of the manipulation tasks.

In these trials, the subject sat in front of a computer screen which showed two screens. The first one was used for stimulus presentation, while the second was used to present the transformed image, as shown in Figure 11. After the baseline period, a stimulus (RHIM, LHIM, or rest) was randomly presented. A total of 15 stimuli (5 for each type) were presented in each trial. A one-second *beep* sound followed a two-second green cross as prestimulus, with a 27–29 seconds interstimulus period. Manipulation tasks were performed according to the result of the classification, and performance was evaluated as the percentage of correctly classified stimuli. The total duration of these trials was considerably longer than in the low-level BCI. This is mainly due to the longer interstimulus period, in which the manipulation tasks took place. Users underwent three sessions on different days, performing five trials in each session.

#### 3.5.2. Uncued Manipulation Trials

For uncued manipulation trials, all stimuli were replaced with the word “Imagine,” and the user freely decided the task to perform, as explained in Section 3.5. A total of 15 stimuli were presented in each trial. The stimulus, prestimulus, and interstimulus duration was the same as in the goal-selection BCI cued manipulation trials (see Section 3.5.1). Immediately after the classification was performed, and before the robot executed the task, the user was asked the type of intended stimulus to emit. In these trials, performance was evaluated as the percentage of coincidences between the intended and the classified stimulus type.

### 3.6. Analysis of Data through P300 Estimation

**Figure 12:**
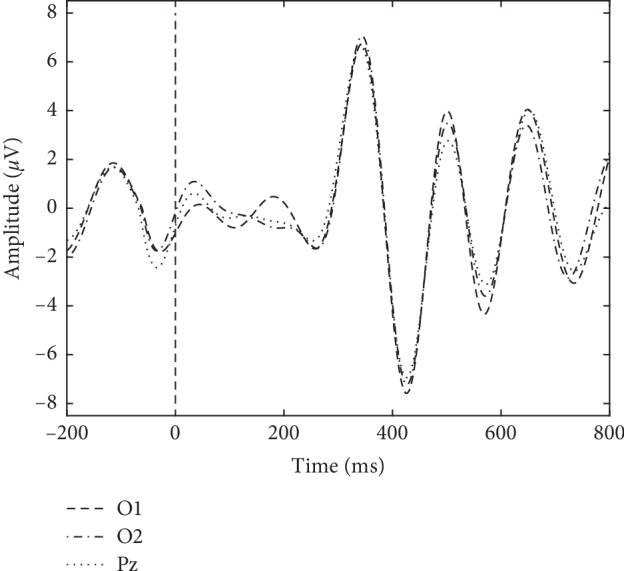
Representation of a P300 waveform calculated for channels O1, O2, and Pz.

Reported assessments of mental fatigue through P300 amplitude and latency can be found in [38] and [19]. In [19], mental fatigue was evaluated through EEG measurements. Participants' P300 were measured during a modified Eriksen flanker task, replacing word stimuli with arrows, before and after performing mental arithmetic tasks. A decreased P300 amplitude and an increased latency were observed after performing arithmetic tasks, when users were mentally fatigued. Statistical analysis revealed the most significant changes in amplitude and latency at channels O1, O2 and Pz, probably as a reflection of visual processing during stimulus presentation of arrows. Similar to the protocol used in [19] to assess mental fatigue, signals were segmented into 1 s stimulus-locked EEG epochs from 200 ms before and 800 ms after stimulus presentation. These epochs were obtained for the presentation of the word “Imagine” during uncued manipulation trials for both the process-control and goal-selection BCIs. For each trial, a representative waveform was obtained by averaging the epochs from all stimuli. Then, the averaged waveforms were band-pass filtered at 1–10 Hz and were used to calculate P300 amplitude and latency. The amplitude was considered as the most positive peak within a 200–500 ms window immediately after stimulus presentation. Latency was obtained as the time this peak appeared. Amplitude and latency values were obtained through this procedure for all trials, sessions, and subjects, in channels O1, O2, and Pz. A representation of an obtained P300 waveform is shown in Figure 12 for these three channels.

In order to examine the differences of mental fatigue within and between users in relationship with the use of our two different BCI schemes, two-way ANOVA tests were performed on all users: one for amplitude and one for latency. In these tests, influence of *trial repetition* (1–5), *channel location* (O1, O2, and Pz), and their interaction were analyzed on both P300 features. The number of replications was considered as three, representing the three uncued manipulation sessions performed by the users. To further analyze mental fatigue related to continuous BCI manipulation, one-way ANOVA (*p* < 0.05) tests were performed on each subject. Six one-way ANOVA tests were performed per subject: three channels (O1, O2, and Pz) × two P300 features (amplitude and latency). These tests were performed in order to find which channel showed significant relationship to the trial repetition factor. Then, amplitude and latency values of all users were compared using the most significant channel from this analysis.

## 4. Results

A preliminary validation of our CGA model and AV algorithm can be found in [26] and [27], respectively; hence, we omit those details here. Therefore, this section shows the results of evaluating the whole system in the context of our BCI implementations for four subjects (two on each BCI type). Performance values were obtained for all subjects in training, cued, and uncued trials, according to the particularities of each experimental protocol. For training trials, performance values correspond to the classifier accuracies shown in Table 3. Performance for cued and uncued manipulation trials was obtained as explained in Sections 3.4 and 3.5. Performance values included in these results represent the average across trials for each session.

### 4.1. Performance of Process-Control BCI

**Figure 13:**
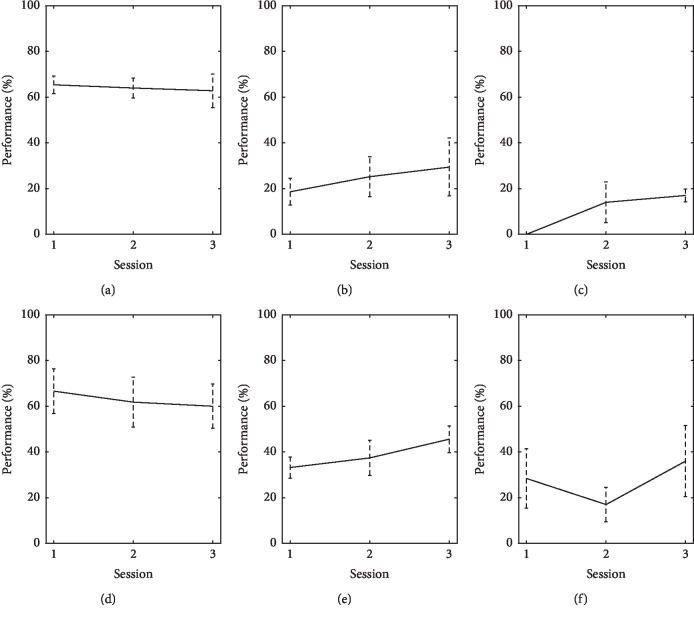
Performance for users *S*_1_ (top) and *S*_2_ (bottom) in process-control BCI during training, cued, and uncued manipulation trials (left, middle, and right columns, respectively). Bars indicate one standard deviation. (a) *S*_1_, training. (b) *S*_1_, cued manipulation. (c) *S*_1_, uncued manipulation. (d) *S*_2_, training. (e) *S*_2_, cued manipulation. (f) *S*_2_, uncued manipulation.

Subject *S*_1_ reached an accuracy level of 65% at its first training session, 64% at the second, and 63% by the third. During cued manipulation trials, performance started at 18% and then increased to 25% and 29% by the second and thirds sessions, respectively. For uncued manipulation trials, the user only moved far from the target at the first session (0%). For the second and third sessions, user *S*_1_ obtained performances of 14% and 17%. Subject *S*_2_ showed a similar behavior to *S*_1_ during training trials, starting at 65% and decreasing to 62% and 60% by the second and third sessions. In cued manipulation trials, performance started at 33% and then increased to 37% by the second session and 45% by the third. For uncued manipulation trials, performance values started at 28% for the first session, then decreased to 17% at the second, and increased to 37% by the third. Results for process-control BCI performance are shown in Figure 13 for users *S*_1_ and *S*_2_, respectively.

### 4.2. Performance of Goal-Selection BCI

**Figure 14:**
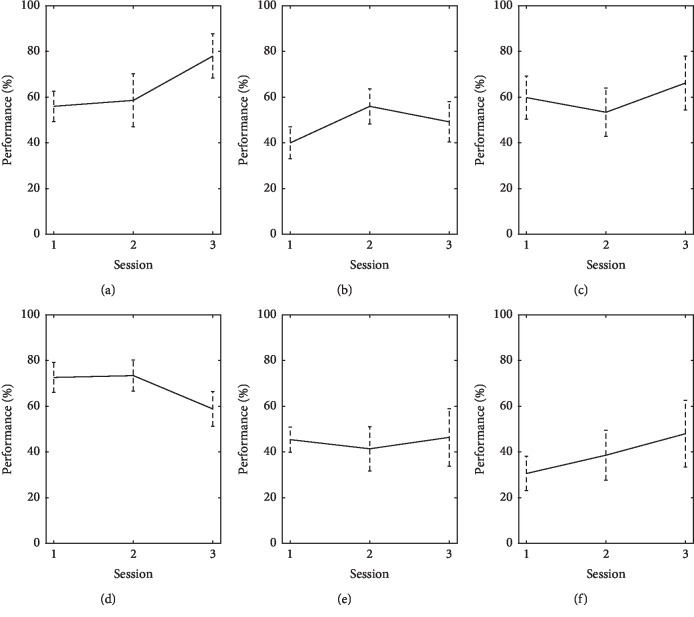
Performance for users *S*_3_ (top) and *S*_4_ (bottom) in semiautonomous goal-selection BCI during training, cued, and uncued manipulation trials (left, middle, and right columns, respectively). Bars indicate one standard deviation. (a) *S*_3_, training. (b) *S*_3_, cued manipulation. (c) *S*_3_, uncued manipulation. (d) *S*_4_, training. (e) *S*_4_, cued manipulation. (f) *S*_4_, uncued manipulation.

Subject *S*_3_ started the training sessions at 56% of accuracy, 58% at the second session, and reached 78% at the third session. Performance for the cued manipulation trials started at 40%, increasing to 56% at the second session and decreasing to 49% by the third. During uncued manipulation trials, performance started at 60% accuracy by the first session, 53% at the second, and 67% by the third. User *S*_4_ obtained performance values of 73% at the first session, 72% at the second, and decreased to 60% at the third. During cued manipulation trials, the subject obtained performance values of 45% for the first session, 41% for the second, and 46% for the third. For uncued manipulation trials, user performance started at 30% and increased to 38% and 48% by the second and third sessions, respectively. Results for goal-selection BCI performance are shown in Figure 14 for users *S*_3_ and *S*_4_, respectively.

### 4.3. P300 Analysis

The results for the two-way ANOVA tests are presented in Table 4. The results for the P300 latency two-way ANOVA showed statistical significance for subjects *S*_1_ (*p*=0.0147) and *S*_2_ (*p*=0.0001) in the trial factor, but no significance was observed for channel and interaction factors. Users *S*_3_ and *S*_4_ showed no statistical significance for any of the analyzed factors. For the P300 amplitude two-way ANOVA, users showed smaller *p* values in trial when compared to channel and interaction. However, our tests did not show statistical significance for any factor or interaction.

The results for the one-way ANOVA tests are shown in Table 5. The results for P300 latency one-way ANOVA showed statistical significance for user *S*_1_ at channel O1 (*p*=0.0476) and for user *S*_4_ at channel O2 (*p*=0.0242). Regarding users *S*_2_ and *S*_3_, *p* values were not significant at any channel. For the P300 amplitude one-way ANOVA, user *S*_4_ showed statistical significance at channel Pz (*p*=0.0019). The tests for *S*_1_, *S*_2_, and *S*_3_ revealed no statistical significance at none of the three analyzed channels.

The results of the performed statistical tests allowed to observe differences between analyzing latency and amplitude. Among all tests, greater changes were found in latency rather than in amplitude. Based on these results, an evaluation and comparison on amplitude and latency values was performed. These values were considered as those corresponding to the channel with the lowest *p* value on the one-way latency ANOVA results. The selected channels were O1 for *S*_1_, Pz for *S*_2_ and *S*_3_, and O2 for *S*_4_.

**Figure 15:**
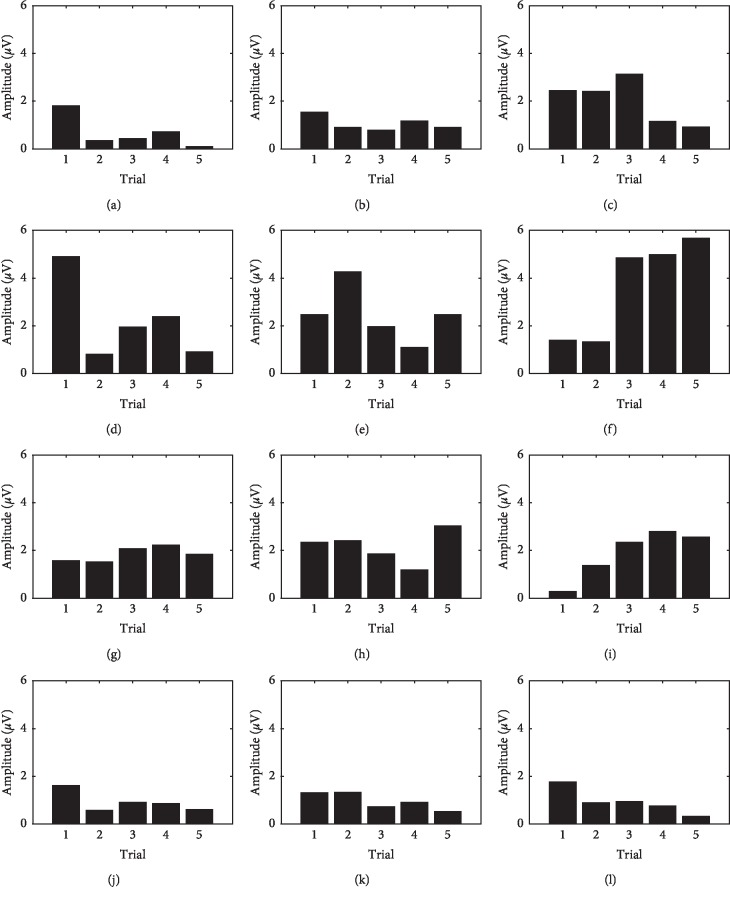
Amplitude of P300 waveform during all trials and experiments for all subjects during uncued manipulation trials. (a) Subject *S*_1_, session 1. (b) Subject *S*_1_, session 2. (c) Subject *S*_1_, session 3. (d) Subject *S*_2_, session 1. (e) Subject *S*_2_, session 2. (f) Subject *S*_2_, session 3. (g) Subject *S*_3_, session 1. (h) Subject *S*_3_, session 2. (i) Subject *S*_3_, session 3. (j) Subject *S*_4_, session 1. (k) Subject *S*_4_, session 2. (l) Subject *S*_4_, session 3.

Amplitude values calculated for all uncued manipulation trials are shown in Figure 15 for each session and user. Users *S*_1_ and *S*_4_ showed a similar behavior: a decreasing P300 amplitude trend in all sessions. In this case, the amplitude observed at the first trial was higher than that of the last one. *S*_2_ showed a decreasing trend as well for the first and second sessions, yet the opposite was observed during the third session. *S*_3_ presented an increasing amplitude trend for all sessions. Here, the amplitude obtained at the last trial was higher than the one at the first trial.

**Figure 16:**
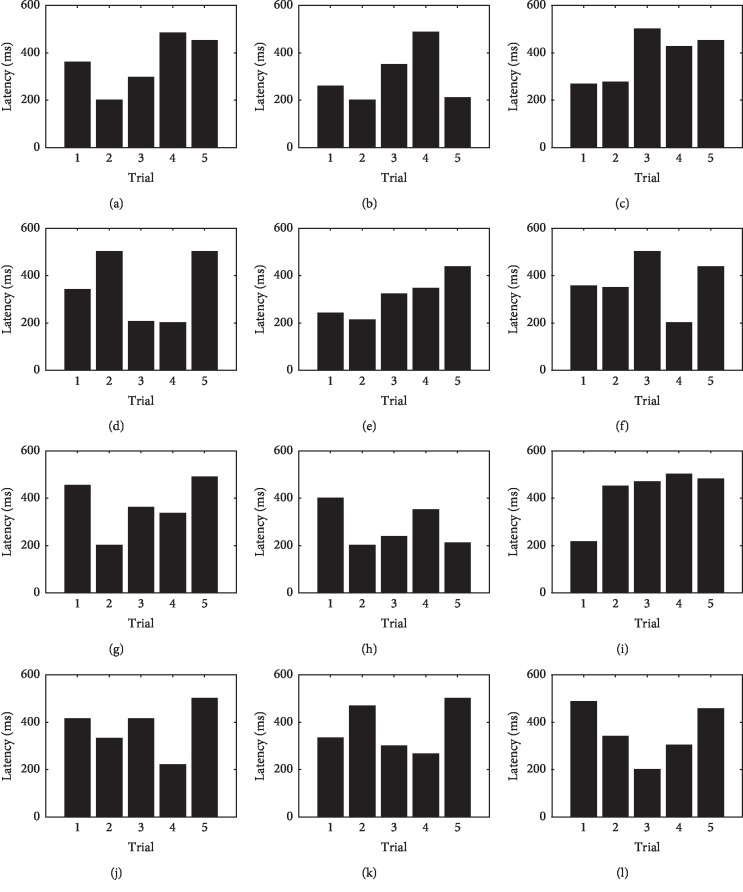
Latency of P300 waveform during all trials and experiments for all subjects during uncued manipulation trials. (a) Subject *S*_1_, session 1. (b) Subject *S*_1_, session 2. (c) Subject *S*_1_, session 3. (d) Subject *S*_2_, session 1. (e) Subject *S*_2_, session 2. (f) Subject *S*_2_, session 3. (g) Subject *S*_3_, session 1. (h) Subject *S*_3_, session 2. (i) Subject *S*_3_, session 3. (j) Subject *S*_4_, session 1. (k) Subject *S*_4_, session 2. (l) Subject *S*_4_, session 3.

Latency values can be observed in Figure 16 for all users and sessions. Subjects *S*_1_ and *S*_3_ showed an increasing P300 latency trend during the first and third sessions. A decreasing trend was observed during the second session for these users. User *S*_2_ presented an increasing latency trend for all sessions. User *S*_4_ showed an increase in latency during the first and second sessions and a decrease at the third.

## 5. Discussion

The implementation and integration of the CGA model and the AV algorithm allowed to successfully design a MI-based semiautonomous BCI for manipulation tasks. When compared against a low-level system, both BCIs were similar in terms of training protocol and control commands; however, the complexity of the executed tasks was different. The semiautonomous goal-selection BCI was superior in task complexity when compared to the process-control BCI, even though both systems used the same control commands as input. While the process-control BCI might be used to perform more general tasks, it demands a continuous awareness state from the user. Its output are discrete low-level commands which in the long run might lead the user to a mental fatigue condition. Although the semiautonomous BCI is goal specific, it requires user attention only during short time periods, making it theoretically less fatiguing. The semiautonomous goal-selection BCI works, in essence, in a more natural way to the user than the process-control BCI. This is because when performing reaching tasks, people think on the main goal and the cerebellum processes the necessary information to successfully achieve it, rather than executing several discrete low-level movements [39].

The selected features for the general classifiers of the users were mainly frontal, central, and parietal electrodes in the *μ* (8–13 Hz) and *β* (13–30 Hz) brain rhythms, which are known to be physiologically involved in the imaginary movement process. The selected channels for the classifiers are consistent with reports of central activity as a reflection of motor cortex contralateral desynchronization during imaginary movement [10] and frontoparietal activation related to control of spatial attention and motor planning during reaching tasks [40, 41].

Even though all users underwent the same training protocol, differences among them were observed. Across training sessions, *S*_1_ and *S*_2_ maintained a relatively constant performance, while *S*_3_ showed a more notorious improvement. *S*_4_ showed a relatively high performance at the first and second session, but it decreased at the third. During cued manipulation trials, all users obtained low performance levels and none of them showed a significant improvement across sessions. *S*_1_ obtained below chance level (33%) performance during all sessions. Performance of users *S*_2_, *S*_3_, and *S*_4_ was in general above chance level, but always remained below 60%. During uncued manipulation trials, users *S*_1_ and *S*_2_ presented the lowest performance values, close to and below chance level. This indicates that these users were faced with difficulty while controlling the process-control BCI. Performance of *S*_3_ and *S*_4_ during uncued manipulation trials was higher (around 40–60%) when compared to *S*_1_ and *S*_2_. Mean performance values across trials of users *S*_3_ and *S*_4_ failed to reach 70% considered as the theoretical threshold for practical MI-BCI use [42]. However, their performance was evidently higher than the one obtained by users performing on the process-control BCI. This might suggest that the designed semiautonomous goal-selection BCI was easier to manipulate than the process-control BCI. Future research will address classification optimization to increase system accuracy and ease of use.

As shown in Table 3, selected channels and frequencies for feature extraction showed changes across sessions for all users. This might suggest that the used channel/frequency selection method is sensitive to intra- and intersubject brain variability. After training trials, a classifier with fixed parameters was selected per subject and used in all BCI trials. Yet, constant adaptation of the classifier parameters is required for optimal operation. Hence, an optimized feature selection algorithm should be implemented to address this issue and increase efficiency in our proposed semiautonomous BCI. Such optimization was out of the scope of our work, but reports on how optimized correlation-based feature selection methods are used in MI-BCIs can be found in [43, 44].

Another efficient approach for feature selection is the partial directed coherence (PDC) analysis, which could help identify relevant channels and features. Recently, a PDC-based analysis was proposed in [45] to identify relevant features for MI tasks, and efficient classifiers were built based on this procedure. Even more recently, a review on EEG classification algorithms highlights Riemannian geometry-based classifiers as promising, as well as adaptive classification algorithms [46]. A simple implementation of an adaptive classifier for MI tasks was described in [47], which showed an encouraging increase on classification accuracy. More novel classifiers based on Riemannian geometry have shown good results in classifying brain-related MI tasks [48].

In regards to our selection of the P300 component to evaluate mental fatigue, such component is not exclusively presented during nonfrequent stimulus, rather its amplitude is enhanced, which makes it a suitable control command for BCIs. P300 amplitude is larger during nonfrequent stimuli, and it is typically used/analyzed based on this argument. However, it has been demonstrated that P300 responses can be observed to both frequent and nonfrequent stimuli [49, 50]. In fact, under a reaction-time regime, P300 is elicited on both predictable and unpredictable stimuli. Task demands increase in this scenario, as users must decide when to respond in a fast and correct manner. This leads to an enhancement of P300, independently of stimulus predictability [49]. In our study, users were instructed to perform MI commands after stimulus presentation of the word Imagine, and P300 components were analyzed immediately after stimulus onset. Although stimulus presentation during uncued manipulation trials could be considered as predictable, P300 analysis holds validity, as it was executed under a reaction-time regime.

Under those conditions, the results of the two-way ANOVA and one-way ANOVA tests showed statistically significant changes in P300 latency for users *S*_1_, *S*_2_, and *S*_4_. Except for *S*_4_, the tests revealed no statistical significance for P300 amplitude. When comparing the amplitude and latency values from Figures 15 and 16, a general trend was found among users: a decrease in amplitude and an increase in latency. These trends in P300 features were presented along trial repetition, that is, after continuous manipulation of the BCI. These changes in amplitude and latency might be related to the generation of mental fatigue, as they are presented after a continuous execution of manipulation tasks through the BCI. It has been shown that a decrease in P300 amplitude and an increase in latency reflect decreased cognitive processing and lower attention levels [19]. Similar results have been found on a P300-BCI evaluated under different levels of mental workload and fatigue [51]. When comparing subjects performing on the same BCI type, the user with the lowest performance exhibited lower amplitude and higher latency values than the user with the highest performance (although it was more evident for amplitude values). This was observed when comparing both *S*_1_-*S*_2_ and *S*_3_-*S*_4_. Subject *S*_3_ showed an interesting behavior: an increasing amplitude trend as well as being the only subject which did not show statistical significance on any P300 test. At the same time, it was the subject with the highest performance values on uncued manipulation trials. A possible explanation to this particular case is that after performing manipulation trials on the BCI, mental fatigue affected differently user *S*_3_ than the rest of the users. This difference in mental fatigue generation was reflected as nonsignificant changes in P300 parameters during the tests as well as higher performance values.

## 6. Conclusions

Two BCI systems, a process-control BCI and semiautonomous goal-selection BCI, were implemented and compared in terms of performance and mental fatigue. The process-control BCI allowed users to perform three-dimensional movements on a robotic arm to reach for a target. The semiautonomous BCI allowed the user to execute manipulation tasks, using the same robotic arm, which include reaching, picking, and placing movements successfully. The increase of task complexity represented by the semiautonomous BCI was achieved without compromising the simplicity of the control procedure, as both BCIs were controlled through MI commands. Users performing on semiautonomous BCI obtained higher performance values when compared to users performing on low-level BCI. The difference in task complexity also represented a difference in the mental fatigue experienced by the users on different systems. A P300 amplitude decrease and a latency increase were found as users performed continuous BCI trials, which is consistent with reports of mental fatigue detection on EEG.

We also present strong evidence of the advantages of semiautonomous BCI in terms of performance and mental fatigue. It is also important to address the potential use of the P300 waveform as an indicator of mental fatigue during BCI testing, training, and evaluation. Techniques to further reduce mental fatigue while using BCI systems might provide an increase in BCI patient acceptance rate as well as a possible path to tackle BCI illiteracy. It is of great importance that the user finds the system as nonfatiguing and easy to use in order to provide a more comfortable and efficient assistance. This also facilitates the user in the process of learning how to control the BCI, which can be used together with different strategies to further personalize the system (see, e.g., a previous work by our group in how to select a feedback modality that better enhances the volunteer's capacity to operate a BCI system [52]).

The development of more advanced semiautonomous BCI systems which provide information about the environment during specific tasks will allow to further enhance performance and usability. Semiautonomous BCIs offer users the possibility to perform more complex tasks in a simple, less fatiguing way. In our system, the integration of the AV and CGA algorithms provided a real-time calculation of the robot's inverse model, offering flexibility to implement more complex object manipulation tasks in a dynamic environment. The use of a higher DOF robotic arm as well as the implementation of object recognition techniques might improve the complexity of the manipulation tasks to be performed while using the same MI commands to control the BCI, ensuring control simplicity to the users.

## Figures and Tables

**Table 1:** Representations of the conformal geometric entities.

Entity	Standard	Dual
Point	*P*=*x*+(1/2)*x* ^2^ *e*_*∞*_+*e*_0_	
Point pair	*Pp*=*s*_1_∧*s*_2_∧*s*_3_	*Pp* ^*∗*^=*x*_1_∧*x*_2_
Line	*l*=*π*_1_∧*π*_2_	*l* ^*∗*^=*x*_1_∧*x*_2_∧*e*_*∞*_
Circle	*c*=*s*_1_∧*s*_2_	*c* ^*∗*^=*x*_1_∧*x*_2_∧*x*_3_
Sphere	*s*=*P* − (1/2)*r* ^2^ *e*_*∞*_	*s* ^*∗*^=*x*_1_∧*x*_2_∧*x*_3_∧*x*_4_

**Table 2:** Parameters for joint angles calculation.

*k*	*α*_*k*_	*β*_*k*_
0	*e*_2_	(*π*_*e*_^*∗*^∧*e*_*∞*_) · *e*_0_
2	(*L*_12_ · *e*_0_) · *e*_*∞*_	(*L*_23_ · *e*_0_) · *e*_*∞*_
3	(*L*_23_ · *e*_0_) · *e*_*∞*_	(*L*_3*e*_ · *e*_0_) · *e*_*∞*_

**Table 3:** Features: EEG channels and frequency range (in Hz) and mean accuracy of LDA classifiers for all subjects and training sessions.

Subject	Session	Features	Accuracy (%)
*S*_1_	1	C4 (11–15), P3 (11–15), C3 (7–11), Fp2 (13–17)	65
	2	C3 (7–13), P3 (9–13), P4 (9–13), P4 (13–17), Cz (9–13)	64
	3	C4 (9–15), C3 (13–17), P3 (23–27), P4 (25–29)	63

*S*_2_	1	C4 (9–13), P4 (11–15), F4 (17–21), F4 (11–15), Cz (11–15)	65
	2	C4 (15–19), C3 (19–23), P4 (21–25), Cz (19–23), FP1 (25–29)	62
	3	C4 (19–23), C4 (17–21), Cz (19–23), P3 (21–25), F3 (19–23)	60

*S*_3_	1	F4 (15–21), F3 (9–11), P4 (9–13), F4 (15–21)	56
	2	C3 (11–15), C4 (7–11), P3 (15–19), P4 (11–15)	61
	3	C3 (9–15), C4 (11–15), P3 (11–15)	78

*S*_4_	1	C4 (9–13), C3 (9–13), P4 (17–21), F4 (19–23), F3 (19–23)	73
	2	F3 (11–15), C4 (7–13), P4 (17–21), P3 (17–21), F4 (19–23)	72
	3	C4 (7–11), C3 (13–17), F4 (17–21), C4 (11–15)	60

**Table 4:** Two-way ANOVA results for P300 latency and amplitude.

	Latency			Amplitude		
Subject	Trial	Channel	Interaction	Trial	Channel	Interaction
*S*_1_	*F*=3.69	*F*=0.25	*F*=0.69	*F*=2.45	*F*=3.08	*F*=0.42
	**p**=0.0147	*p*=0.782	*p*=0.6994	*p*=0.0676	*p*=0.0609	*p*=0.8969

*S*_2_	*F*=9.33	*F*=0.13	*F*=0.1	*F*=1.26	*F*=0.48	*F*=0.14
	**p**=0.0001	*p*=0.8816	*p*=0.999	*p*=0.3074	*p*=0.6217	*p*=0.9970

*S*_3_	*F*=0.03	*F*=0.59	*F*=0.44	*F*=0.83	*F*=0.01	*F*=0
	*p*=0.9983	*p*=0.5604	*p*=0.8891	*p*=0.5191	*p*=0.9924	*p*=1

*S*_4_	*F*=1.05	*F*=1.5	*F*=0.3	*F*=1.81	*F*=0.17	*F*=0.13
	*p*=0.4003	*p*=0.24	*p*=0.9589	*p*=0.1534	*p*=0.8405	*p*=0.9972

Bold values highlight those for which *p* < 0.05.

**Table 5:** One-way ANOVA results for P300 latency and amplitude on channels O1, O2, and Pz.

	Latency			Amplitude		
Subject	O1	O2	Pz	O1	O2	Pz
*S*_1_	*F*=3.54	*F*=0.76	*F*=0.55	*F*=1.79	*F*=1.61	*F*=0.82
	**p**=0.0476	*p*=0.5767	*p*=0.7055	*p*=0.2066	*p*=0.246	*p*=0.5421

*S*_2_	*F*=2.1	*F*=2.13	*F*=2.5	*F*=0.3	*F*=0.23	*F*=0.1
	*p*=0.1554	*p*=0.1518	*p*=0.1091	*p*=0.8705	*p*=0.9171	*p*=0.9806

*S*_3_	*F*=0.37	*F*=0.12	*F*=0.54	*F*=1.31	*F*=0.13	*F*=0.41
	*p*=0.8238	*p*=0.9731	*p*=0.7089	*p*=0.3322	*p*=0.9687	*p*=0.8043

*S*_4_	*F*=1.68	*F*=4.52	*F*=2.45	*F*=2.99	*F*=2.27	*F*=9.5
	*p*=0.2311	**p**=0.0242	*p*=0.1138	*p*=0.0728	*p*=0.1336	**p**=0.0019

Bold values highlight those for which *p* < 0.05.

## Data Availability

The electroencephalography datasets used to support the findings of this study are available from the corresponding author upon request.

## Supplementary Materials

Supplementary Materials Video #1 (file: VID_20180629_133018082∼2.mp4): this video shows the operation of the semiautonomous BCI system, specifically the part of the robotic arm performing the process of picking the disk and replacing it over one of the target areas. The pick-and-replace process is repeated once the position of all items is changed randomly. Incidentally, the robot places the disk in the green (left) target in both occasions, as that was the one selected by the user in the two trials shown here. Video #2 (file: VID_20180629_133317483∼2.mp4): this video shows the user, in a contiguous room, interacting with the BCI system. The brain activity of the user is measured with the EEG system, and a stimulus is provided through a red arrow in the screen which indicates the MI that the user has to perform. Also, the user receives video feedback of the robot movement. Video #3 (file: VID_20180629_133853176∼2.mp4): this video shows the screen of the computer that processes all the data that are being acquired both by the EEG system and the AV algorithm. The screen also shows the CGA model that is adjusted on the fly based on the positions of the items detected by the AV algorithm and the selected target area where the disk will be placed.
